# Considerations for Pain Assessments in Cancer Patients: A Narrative Review of the Latin American Perspective

**DOI:** 10.7759/cureus.40804

**Published:** 2023-06-22

**Authors:** Joseph Pergolizzi Jr, Jo Ann K LeQuang, Flaminia Coluzzi, Peter Magnusson, Argelia Lara-Solares, Giustino Varrassi

**Affiliations:** 1 Pain Medicine, NEMA Research, Inc., Naples, USA; 2 Healthcare Policy, NEMA Research, Inc., Naples, USA; 3 Medical and Surgical Sciences, Sapienza University of Rome, Rome, ITA; 4 School of Medical Sciences, Örebro University, Örebro, SWE; 5 Pain and Palliative Care, National Institute of Medical and Nutritional Sciences, Mexico City, MEX; 6 Pain Medicine, Paolo Procacci Foundation, Rome, ITA

**Keywords:** pain assessment tools, cancer in latin america, spiritual pain, pain intensity, pain assessment, latin american cancer patients, cancer pain assessments, cancer pain, brief pain inventory

## Abstract

Cancer incidence in Latin America is lower than in Europe or the United States but morbidity and mortality rates are disproportionately high. A barrier to adequate pain control is inadequate pain assessment, which is a relatively easy and inexpensive metric. The objective of this narrative review is to describe pain assessment for cancer patients in Latin America. Cultural factors may influence pain perception, including contextualizing pain as noble or natural suffering and aspects of what is now called “spiritual pain.” Unlike other painful conditions, cancer pain may be strongly associated with existential fear, psychosocial distress, anxiety, and spiritual concerns. Pain assessment allows not just quantification of pain intensity but may elucidate pain mechanisms involved or psychosocial aspects that may color the pain. Many current pain assessment instruments capture only pain intensity, which is but one aspect of the pain experience; some have expanded to include functional assessments, mental health status evaluations, and quality of life metrics. A quality-of-life assessment may be appropriate for cancer patients since chronic pain can severely impact function, which can in turn create a vicious cycle by exacerbating pain. The incidence of cancer in Latin America is expected to increase in the ensuing years. Better pain assessment and clinician education are needed to help manage pain in this large and growing patient population.

## Introduction and background

While cancer incidence in Latin America (Central America, Mexico, South America, and Caribbean nations) is lower than the rates in Europe or the United States (163 vs. 264 vs. 300 per 100,000, respectively), the morbidity and mortality burden of cancer in Latin America is disproportionately large [1]. About 70% of all people with cancer report pain, which is inadequately controlled in about half of this population [2]. This pain may be due to the cancer or related to cancer treatments. Poor pain control may result in longer hospital stays, loss of productivity, functional deficits, shortened life expectancy, and greater utilization of healthcare resources [3, 4]. Among cancer survivors or those living with managed disease, 20% to 50% continue to experience pain after treatment has stopped [5]. While it has been estimated that about 90% of cancer patients or survivors should be able to obtain some degree of pain relief, the primary barrier to adequate pain control is the failure to assess pain accurately and regularly [6,7]. The objective of this narrative review was to describe pain assessment for cancer patients in Latin America with the goal of offering information that may guide clinicians in assessing pain in this population and raising specific considerations for Latin American clinicians.

Methods

This is a narrative review. The PubMed database was searched for the keywords “cancer pain assessment + Latin America” and there were 21 results (no delimiters were used). The same terms were searched in Google Scholar. The bibliographies of these articles were searched as was general information about specific cancer pain assessment tools. This is a narrative rather than a systematic review intended to offer insight into pain assessment in cancer patients with a specific emphasis on Latin American nations.

## Review

A global perspective on the evolution of pain assessment in cancer patients

Pain assessment has evolved since 1986 when the World Health Organization (WHO) first presented its now iconic “pain ladder” and recommended treating cancer pain based on pain intensity [8]. As pain was better understood as a biopsychosocial experience, its impact on daily life and patient function was also considered [9]. Awareness of multiple pain mechanisms and different types of pain allowed for better pain assessments and treatments [10]. A holistic perspective recognizes that untreated pain may have adverse influences on health because it interferes with restorative sleep, appetite, mood, and energy levels [11]. In noncancer patients, chronic pain is often defined temporally by enduring for three to six months, but such definitions may not be helpful for cancer patients with very short life expectancies [12]. Many factors can influence the pain symptoms cancer patients experience: the type of cancer, its relative progression, treatment types, sex(women report more pain and more intense cancer pain than men), outpatient status (outpatients tend to have less intense but longer-duration pain than inpatients), and the presence of metastatic disease [12]. Cancer pain can be complicated by sudden-onset exacerbations of pain known as “breakthrough pain,” which requires rapid-onset analgesia [13, 14]. Cancer patients often have multiple pain sites with different mechanisms and present with mixed pain syndromes [15]. Even a single pain site may have multiple pain mechanisms. and a neuropathic component to pain is not uncommon in cancer patients [16]. A variety of validated pain measurement assessment tools have been developed with the goal of allowing clinicians or patients themselves to rapidly but accurately quantify their pain levels. While nearly all of these tools evaluate pain intensity, some have expanded to include functional assessments, mental health status evaluations, and quality of life metrics [17]. There is no broad consensus as to how to measure cancer pain and no universally accepted pain assessment instrument to evaluate pain in cancer patients [17]. Understanding the underlying pain mechanism(s) can guide prescribing choices. Many of the pain assessment instruments used today were developed for acute noncancer pain and best capture the intensity of the pain, which is only one aspect of the pain experience [18]. Cancer pain and noncancer pain have many similarities, but the context in which that pain occurs differs, which may affect pain perception, pain levels, and coping strategies. Cancer can be a terminal condition, and palliative pain control strategies differ radically from techniques used to manage pain in individuals who are expected to recover fully and even those who are expected to live for years with managed disease.

Assessing pain in cancer patients

Cancer can be a distressing and overwhelming diagnosis, which can affect both pain levels and how the patient contextualizes pain. Cancer can be a prolonged and brutal experience, and patients may face different physical, mental, and psychological challenges at various junctures. Pain control must consider the prognosis as well since those expected to make a full recovery will have different pain control objectives than a terminally ill patient. Cancer pain can wax and wane, causing marked variations in cancer pain assessments over time. Cancer pain may be caused by the underlying disease, by the anticancer treatments or surgeries; in some cases, the pain may be unrelated to the cancer [19].

Despite the fact that analgesic options are available, cancer patients may downplay their pain or even deny it [20]. The reticence to discuss real pain may be caused by any of several reasons that the patient may not be able to specifically articulate, such as fatalism, an attitude that complaining is a sign of weakness, a fear that worsening pain means worsening disease, and a belief that talking about pain could distract the clinical team from fighting cancer [21]. Pain assessments are often associated with prescriptions for opioid analgesics, and some patients resist opioids out of concern about addiction, side effects, or the idea that tolerance will build up and that pain relievers will not work when they most need them [22, 23].

Cognitive deficits, the disease state, sedation, language barriers, problems speaking, or other reasons may impair communications, and even make patient pain self-reports impossible. In such cases, observed physiological measurements may have to replace the patient’s evaluation of pain. Although not the optimal form of pain assessment, there are assessment tools available for signs of pain. The Behavioral Pain Scale and the Checklist of Nonverbal Pain Indicators (CNPI) rely on body postures, behaviors, facial expressions, and vocalizations to help measure pain [24, 25]. Reliable biomarkers for pain assessment remain unknown but are an important unmet medical need [26].

When cancer patients are asked to rate their pain intensity, they may be asked by assessment tools to describe pain at that particular moment, their pain over the last 24 hours, or even over the last week. Respondents are sometimes asked to describe the worst pain that occurred over a past window of time or the average pain level of the preceding week. Since memory is subject to inaccuracy, using recollections about past pain, particularly far, distant recollections, can introduce inaccuracy into pan measurement [27]. Cancer pain can change markedly with the course of the disease and treatments. Note also that pain measured at rest or during motion can be different [27].

The context of cancer pain is important to consider, as cancer is a severe, life-altering, and potentially life-threatening disease [28). Cancer pain, more than other types of pain, is characterized by sudden flares of pain against the ambient background of pain; these breakthrough episodes can be extremely distressing, debilitating, and challenging to treat [13]. Furthermore, pain can worsen or abate over the natural course of the illness, for example, it may worsen during chemotherapy and abate if the patient experiences remission. Cancer patients may suffer from anxiety, dread about the future, catastrophizing thoughts, and justifiable concerns about their families and finances. All of these things can influence how the patient contextualizes and perceives pain, so a holistic assessment is warranted [28].

Cultural factors from Latin America that may influence cancer pain perception

Latin America is not a monolithic cultural entity, but some general observations may play a role in how Latin Americans, in general, experience and describe pain. Latin America has a historic mistrust of agencies representing international health concerns or a global health agenda [29]. In Latin America, there is overall a relatively low level of health literacy, combined with social preferences for traditional and herbal medicines [30, 31]. Among the reasons for using traditional medicines include cultural familiarity, lack of access to Western medicine, and the popular assumption that natural remedies are safer than pharmaceutical products [32]. However, in Latin America, both traditional and modern medical approaches are often combined, particularly to manage complicated diseases such as cancer [32].

Latin American cultures often accept pain as a natural part of living, rather than seeking its medical control [33]. Traditional Latin American social roles for strong, uncomplaining men and quiet, self-sacrificing women, can run counter to aggressive pain management strategies [33]. Latin American cultures place a high value on work and productivity and stress strength in the face of pain and other adversity [34].

Religious affiliation and spirituality can affect how individuals experience and contextualize cancer, pain, suffering, and death [35]. As end of life nears, spirituality and religious convictions emerge as powerful forces, so much so that palliative medicine now recognizes the concept of “spiritual pain,” even if there is no consensus definition for it [36-38]. Spiritual pain has been described as a profound but non-physical pain related to the awareness of death together with a multifaceted sense of loss: loss of self, loss of control, loss of purpose, and loss of relationships [39, 40]. Spiritual pain is deeply colored by religious affiliation and culture as well as personal convictions. In a survey of 100 palliative oncology patients in the United States (88% Christian, 8% other religions, 4% atheists) 100% of respondents said that their spiritual beliefs were a source of strength and comfort for them and 44% reported feelings that could be described as spiritual pain and 60% said their current cancer treatment did not support their spiritual needs [36, 37]. While there are no studies on this subject, Latin American cancer patients also experience spiritual pain, which may be influenced by both Catholic principles regarding the value of suffering as well as folk and traditional ideas. Indeed, the English word “pain” is not exactly equivalent to the Spanish word “dolor,” which carries with it a connotation of emotional pain, loss, and sadness together with physical pain [41]. This emotional dimension of physical pain often goes unrecognized by medical teams [36, 37]. In cancer patients at end of life, spiritual pain has been associated with reduced quality of life [42]. In a meta-analysis of palliative care patients in Latin America, belief in a Supreme Deity was consistently reported as both a source of strength and hope, but also as a factor to help resign oneself to death [35].

The degree to which patients participate actively in their care has some Latin American distinctives. A palliative outcome scale test was administered to 91 cancer patients in Cuba and found that only 41% knew they had cancer. This is in keeping with a widespread Cuban practice of not informing cancer patients of their diagnosis [43]. Cancer patients in Latin America have diverging views on how involved they wish to be in their own cancer care. In a survey of 387 cancer outpatients from Argentina, Chile, Guatemala, and Hispanic cancer patients in the United States, 48% said they preferred shared decision-making with their families and clinical team, but 21% stated they favored a passive approach of letting families and the medical experts take charge [44]. In a study of Hispanic cancer outpatients in New York (n=271), 65% of respondents did not know their cancer stage, 38% did not know if they had metastatic disease, but only 15% said they wanted more information about their condition [45].

Pain assessment tools

Most pain assessment tools were designed to evaluate acute pain but have been utilized with good success for chronic noncancer pain. Many pain assessment tools were not designed for cancer patients but still can be effectively used in that population [46]. For cancer pain, a more comprehensive and holistic evaluation is likely needed to fully assess the patient’s pain experience [47]. The initial pain assessment should include a complete patient history along with a physical examination. The patient should be asked about all pain sites and each one considered individually as pain mechanisms at one site may be different from pain mechanisms at other sites [10]. Going from one pain site to the next, the patient should be asked about a history of the pain: when it started, what caused it, and if certain things seem to make the pain better or worse. It is important to consider the patient’s comorbidities and pre-existing pain syndromes as well as current cancer treatments, cancer prognosis, mental health status, and adverse life consequences associated with cancer or pain, such as deteriorating quality of life, worry about the future, financial loss, family situation, living conditions, or other issues. Using assessment metrics, the clinical team should then regularly assess and document pain [46, 47]. This information will help guide prescribing choices for pain control, but cancer pain treatment goes beyond the scope of this article.

There is no clear consensus as to the best pain assessment tool to be used and sometimes more than one tool may be used. A good pain assessment tool is one that is easy to use, quick to complete, and that the clinical team and patient readily understand. Scores should be quantified for easier assessment and an ability to analyze trends. A short description of some of the major pain assessment instruments follows, in alphabetical order.

Brief Pain Inventory

The Brief Pain Inventory (BPI) uses the eleven-point numerical rating scale as its basis but presents it as a questionnaire that can be filled out by the patient or administered in an interview. The BPI takes about five minutes to complete and it asks patients to describe pain at the time of the survey and to recollect the least, most, and average pain over the past 24 hours [27]. The BPI offers two scales: one for the sensory dimension measuring pain intensity, the other for a reactive dimension in terms of how the pain has interfered with the activities of daily living [9]. There are both short and long forms of the BPI. A Spanish version of the short form (BPI-S) has been validated for both scales [48]. The BPI has also been translated into Portuguese [46]. Although not developed for cancer patients, BPI has been widely used in this population and has been validated for patients with bone metastases, breast cancer, and pain following cancer surgery [46, 49].

Cancer Pain Prognostic Scale

The Cancer Pain Prognostic Scale (CPPS) was not designed to assess pain but instead provides a way to assess effective analgesia in cancer patients with moderate to severe pain. CPPS evaluates pain in physiologic, sensory, affective, cognitive, behavioral, and sociocultural dimensions; pain is evaluated in terms of pain characteristics, physical symptoms, psychological distress, quality of life, and patient characteristics. In evaluations of the CPPS, it was found that predictors of pain control differed over time, suggesting that there are both short-term and long-term cancer pain syndromes [50]. This is a predictive tool rather than an assessment tool, but it may be valuable in the care of cancer patients with pain.

Clinically Aligned Pain Assessment

The Clinically Aligned Pain Assessment (CAPA) is a checklist that clinicians can use to guide a short patient interview, prompting the patient to describe their degree of comfort, their pain and the functional limitations it imposes, and how pain affects sleep. The patient is not required to assess the pain with words or numbers, although CAPA asks if the pain has improved or worsened over time. The clinician-interviewer codes the conversational responses to arrive at a numerical score [51]. CAPA was designed for acute pain and may be used in place of older numeric rating systems [52]. In a study at two hospitals interviewing 63 nurses and 95 patients, both nurses and patients were more satisfied with the CAPA than the numerical rating scale and felt it offered a more holistic view of their pain experience [53].

Doleur Neuropathique en Quatre Questions

The Doleur Neuropathique en Quatre Questions (DN4) instrument is a four-item questionnaire to aid clinicians in distinguishing between neuropathic and non-neuropathic pain syndromes. The DN4 relies on patient self-reports and requires physician input. In a study of 160 patients, the DN4 had good inter-rater reliability and could effectively differentiate neuropathic pain from other types of pain [54]. In a study of 392 patients using the DN4, the instrument had 82% sensitivity and 81% specificity [55]. Sensitivity meant that the DN4 was 82% accurate in identifying neuropathic pain and specificity means that is correctly precluded other forms of pain that might be mistaken for neuropathic pain 81% of the time. Although not designed for cancer patients, the DN4 is often used in this population. A validated Spanish version is available, and the DN4 has been translated into Portuguese [56, 57].

Edmonton Instruments

There is an Edmonton Pain Staging System (EPSS), an Edmonton Symptom Assessment Scale (ESAS), and an Edmonton Classification System for Cancer Pain (ECSCP). These pain scales differ from other evaluation tools in that their goal is to predict effective pain control rather than assess pain [50]. Patients who score as Stage I are significantly more likely to achieve good pain control than those in Stages II or III [58].

The EPSS was created to stage pain in such a way that the key features of that pain might help guide prescribing choices for optimal pain relief. Although not designed expressly for cancer patients, the EPSS has been validated in hospice patients with cancer [50]. In a study of 276 cancer patients, the EPSS had a 93% sensitivity and 46% specificity. Although specificity was low, the scale could still help stratify patients for pain control [50, 58]. A revised version of the EPSS has been released (rESS) which evaluates mechanisms of pain, the presence and severity of incidental pain, psychological distress, addictive behaviors, and cognitive function [59]. In a comparison of the original EPSS with the rESS, both instruments could predict pain control well, but the rESS was better able to predict stable pain control [60].

The ECSCP was specifically designed as an international assessment tool for cancer pain. A multicenter study in several European nations, Canada, and Australia (n=1,070) found the ECSCP was able to describe pain features reliably and effectively across multiple different cultures [61]. In another study of 1,050 adult cancer patients from 11 centers in Canada, the ECSCP was effective in identifying key features of cancer pain. In this study, 87% of cancer patients reported pain, of whom 25% had neuropathic pain or pain with a neuropathic component. An interesting finding from this study was that significant predictors for neuropathic pain in cancer patients were younger age (<60 years) and the presence of incidental pain [62].

A diagnostic algorithm for breakthrough pain has been incorporated into the ECSCP which helps predict the incidence of breakthrough pain episodes and whether flares were nociceptive versus neuropathic or mixed pain syndromes [63]. In a study of 277 cancer outpatients in Spain, breakthrough pain was reported by 39% of all patients and occurred more often in men than women (64% vs. 36%, respectively). Using this algorithm, the ECSCP detected 488 different types of breakthrough pain, of which 50% were neuropathic or had a neuropathic component [63].

The FACES Scale

Originally developed to help pediatric patients self-report acute pain, the Wong-Baker FACES scale (FACES) is based on cartoon-like drawings of facial expressions to indicate various pain levels [64]. In a study of 120 pediatric patients in an emergency room for an acute painful condition, the FACES scale had excellent correlation with the visual analog scale and pain scores did not vary among populations by sex, age, or pain location [65]. In a prospective study of 197 children, FACES correlated well with the visual analog scale and children did not mistake the facial expressions of pain for expressions of fright [66]. The original scale had seven faces which were later amended to six [67]. The FACES tool is generally recommended for use in patients aged four and older, but it also has utility among geriatric patients and those with communication problems [68-70]. A Catalan-Spanish version of the FACES scale was validated among Catalan children who preferred it to a colored analog scale, where pain levels corresponded to different colors [71]. The FACES scale can be easily converted to a numerical rating scale for documentation purposes.

Functional Assessment of Cancer Therapy

The Functional Assessment of Cancer Therapy (FACT) instrument is a questionnaire for cancer patients self-reporting their holistic quality of life [72]. There are variations of the FACT tool, including a general format, FACT-G, and a form specifically for breast cancer patients (FACT-B), which was validated and deemed suitable for use in clinical trials as well as clinical practice [73]. The FACT-M test for melanoma has likewise been validated [74]. In a review of studies on FACT-G, it was found that its overall reliability was 0.88 but there was variation in responses by ethnicity and cancer type [75]. The FACT-G was validated and found effective for use in Latin American cancer patients [76].

ID-Pain

The ID-Pain instrument is a six-item survey that patients complete to aid in the differentiation of neuropathic pain from other painful symptoms [55]. The survey can be entirely completed by the patient without clinician input or supervision. It was not designed for cancer patients, but a study of 240 breast cancer patients validated that it had a 0.72 predictive value for neuropathic pain compared to diagnosis by a clinician [77]. A validated Spanish version was found to be a reliable self-assessment tool for pain patients [78].

Leeds Assessment of Neuropathic Symptoms and Signs

The Leeds Assessment of Neuropathic Symptoms and Signs (LANSS) combines a patient report of sensory symptoms with a bedside examination [79]. Since approximately 39% of cancer patients have some degree of neuropathy, neuropathic pain metrics can be very helpful to guide analgesic choices. Suggestive symptoms, such as burning or shooting pain, numbness, “pins and needles,” and so on, do not specifically confirm neuropathic pain, so an assessment is needed [80]. A shorter form of LANSS intended for patient self-report (S-LANSS) was validated as a reliable metric for neuropathic pain assessment [81]. Note that the LANSS instruments were not designed for cancer patients, but have been shown effective in evaluating neuropathic pain in cancer patients [82]. The LANSS measurement systems were validated in Spanish and Portuguese [83-85].

McGill Pain Questionnaire

The McGill Pain Questionnaire (MPQ) offers both long and short forms and assesses pain in terms of the sensory, affective, and emotional qualities of pain as well as the duration of painful symptoms. Using numerical ratings, patients self-report their pain experiences, which are combined into a total numeric score [27]. The bulk of the MPQ involves 15 descriptors that the patient rates on a four-point scale, with 0 least and 3 most severe. The MPQ also has a numeric rating scale used to capture pain intensity [86]. The short form MPQ has been reported as being effective and of value in assessments of chronic cancer pain [87]. The short form MPQ is available in Portuguese, Spanish, and several other languages [88-90].

Mini-Mental Adjustment to Cancer Scale

The Mini-Mental Adjustment to Cancer (Mini-MAC) instrument does not assess pain but does evaluate cancer-coping attitudes that can play an important role in analgesic effectiveness [91]. Mini-MAC quantifies the patient’s level of adaptive and coping skills based on five attributes: a fighting spirit, fatalism, helplessness/hopelessness, anxious preoccupation, and cognitive avoidance [91]. In a study of 1,187 cancer outpatients in Poland, it was found that Mini-MAC scores varied by sex, the presence of metastasis, and whether or not the patient was currently undergoing chemotherapy [92]. The Mini-MAC instrument has been translated into many languages, including Spanish and Portuguese, and the Portuguese versions have been validated for use in palliative cancer patients [93-96]. 

Numerical Rating Scale

One of the most familiar pain assessment tools is the numerical rating scale (NRS), which asks patients to self-report their pain level on an eleven-point scale, with 0 as no pain at all and 10 as the worst pain imaginable. Pain scores of 1 to 3 are considered mild pain; scores of 4 to 6 are moderate pain; and pain scores of 7 to 10 are considered severe pain. These groupings map onto the verbal rating scale where patients are asked to rate their pain as mild, moderate, or severe [27]. This assessment only measures pain intensity, but it is easy to use, requires no special instrument or props, and is widely understood even by patients who had not used it before. No special translations are needed.

The NRS can also be expanded to use numbers from 0 to 100, which may work better in certain cases, allowing for more nuanced assessments [97]. Although not particularly sophisticated or complicated, the NRS correlates well with other pain assessment measures and is feasible for use in many settings, including telehealth [97-100].

Pain Coping Strategies Questionnaire

The Pain Coping Strategies (CSP) questionnaire is a 27-item survey that covers six domains of coping mechanisms: distraction, catastrophizing, ignoring pain, distancing from pain, self-statements of coping, and prayer [101]. This assessment tool was not designed for cancer pain although it has been found effective in cancer patients [102]. The CSP does not assess pain; rather, it is used to determine how well patients with pain will be able to cope with their situation. In a study of 1,187 cancer outpatients using the CSP, it was found that the patient’s level of education, income, and use of radiotherapy were the most significant predictors for achieving pain management goals [102]. A short form of the questionnaire is in development for use in pediatric patients with chronic pain [103]. Because this scale deals with coping mechanisms, it is vulnerable to cultural influences; a Spanish version of the CSP is available [104].

painDETECT

The painDETECT questionnaire (PDQ) is a screening tool to distinguish neuropathic pain from other types of pain in patients with chronic low back pain, a very prevalent source of chronic pain [105]. This questionnaire has been translated into numerous languages and used in patients with other conditions, including malignancies [106]. In a study in which 291 pain patients were evaluated by both a physician and PDQ, the PDQ instrument had an 80% sensitivity and 55% specificity versus a physician assessment [107].

Verbal Rating Scale

The verbal rating scale (VRS) is a patient self-report given in response to a clinician asking the patient to rate pain on a four-point scale, described as no pain, mild pain, moderate pain, or severe pain. Other adjectives may be used and the VRS can be readily translated onsite. The VRS is most typically used to rate pain experienced at that moment, the worst pain experienced over the past 24 hours, or the average pain over the past 24 hours [27]. The VRS is frequently used to measure acute pain, but it can be used for chronic pain in cancer as well. Although very streamlined and simple, the VRS correlates well with other pain assessment metrics [99, 108]. The VRS is fast and easy to administer. One limitation of the VRS is that the patient may struggle to select the right pain category if the pain is on the cusp of categories, for instance, whether the pain is at the high end of “moderate” or the low end of “severe.” Another limitation of the VRS is that patients may have different ideas about what constitutes each pain category.

Visual Analog Scale

The visual analog scale (VAS) is a visual aid in the form of a picture of a straight line with “no pain” at the terminus at the far left end and “worse pain imaginable” at the far right end [97]. The patient then marks the pain level at some point on the line. The VAS can be modified by adding numbers and tick marks (for example using numbers) or words beneath the line (mild, moderate, severe), both of which are also called graphic rating scales. Although the VAS and graphic versions are widely used, some patients, especially the elderly, have difficulty understanding them [109].

Challenges in pain assessment for cancer patients

Compared to noncancer pain, cancer pain is more likely to be associated with existential fear, psychosocial distress, anxiety, financial stress, and spiritual concerns [110, 111]. Cancer pain may also bring with it a sense of loss, spiritual pain, and family or household troubles. These things can impact pain scores and pain perception. Among Latin Americans, advanced cancer care may be inaccessible, opioid analgesics limited or not available at all, and cancer care managed in large part by the family rather than in a hospital setting. These factors can color the pain experience in extreme and adverse ways. Palliative care is relatively new in Latin American oncology settings but is of increasing importance. Palliation is now offered earlier in the disease and not just at end of life [112]. Another challenge in pain assessment is that cancer patients may often present with a variety of somatic symptoms related to their disease as well, such as fatigue, anorexia, lethargy, and tremors [113]. Finally, many cancer patients have pre-existing pain syndromes from unrelated conditions which can also greatly trouble them as they are being treated for cancer.

Pain assessment tools rely on the ability of the patient to communicate with the clinical team, but cancer patients may be sedated, suffer cognitive impairments, or otherwise not be forthcoming about their pain [110]. While cancer patients may not wish to discuss their pain for any number of reasons, Latin American patients may have cultural reasons to avoid complaining about pain or may place more faith in traditional cures for pain than treatments obtained from a physician.

A quality of life (QoL) assessment may be appropriate for cancer patients since chronic pain can have a devastating impact on everyday life. However, terminally ill cancer patients were found in one study to have similar or even better QoL than chronic noncancer pain patients [114]. Some of the best-known QoL assessment tools include the Short Form Health Survey (SF-36), the World Health Organization Quality of Life Questionnaire (WHOQOL-BREF), the European Organization for Research and Treatment of Cancer Quality of Life core cancer (EORT-QOL- C30), the Functional Assessment of Cancer Therapy (FACT)/Functional Assessment of Chronic Illness Therapy (FACIT) [115, 116]. Some QoL instruments were created for a specific type of cancer, such as breast cancer or prostate cancer. Validated Spanish versions exist for SF-36 and EORT-QOL-C30, and the 39-item Evaluating the Measurement of Patient-Reported Outcomes (EMPRO) questionnaire [117]. Among these instruments, there is no one that is clearly superior to the others [118]. 

Palliative care

As palliative care emerges in Latin America as a new and distinct medical service, the values associated with a palliative paradigm may make palliation in these nations different from palliation in the United States and Europe [35]. While pain control is an important part of palliative care in the United States and Europe, Latin American caregivers may sometimes believe that pain is somehow noble, spiritual, or such an essential part of the human experience it should be endured rather than treated. In a meta-analysis of Latin American familial cancer caregivers, it was found that some regarded palliation as the abandonment of their loved one by the healthcare system, while others saw it as a humanized way to navigate end of life [35].

In a survey of 387 cancer caregivers in Latin America versus Hispanic caregivers in the United States, most respondents overall in both geographies preferred a shared decision-making model for palliation that would allow them to work with the terminally ill patient and the clinical team to make end-of-life decisions [119]. The location where death occurs may also play a role in pain control and palliation. In a study of 2,994,685 deaths in 12 nations of Latin America (Argentina, Brazil, Chile, Colombia, Costa Rica, El Salvador, Guatemala, Ecuador, Mexico, Paraguay, Peru, and Uruguay) from 2016 to 2018, it was found that 58% of deaths were in hospitals while 31% occurred at home, but there were differences among countries. For example, Guatemala had the highest proportion of home deaths, and Brazil had the lowest (68% vs. 20%, respectively). People in rural areas, older age, and lower educational status were all associated with dying at home in Latin America [120]. It should be noted that this study considered all-cause mortality (not just cancer) and was conducted prior to the COVID-19 pandemic. Dying at home was less frequent in Europe and North America, with rates ranging from 13% for home deaths in Canada compared to a high of 41% in Italy for cancer patients [121]. In Latin America, death in a hospital was more likely for cancer than non-cancer patients, although the reverse is true for cancer patients in Europe and North America. This is likely due to limited palliative care resources in Latin America and the fact that hospital resources are not abundant for Latin Americans at end of life [120],

Discussion

Pain assessment is a crucial factor in effective pain control in cancer patients, but it goes beyond the scope of this article to describe analgesic regimens for cancer patients. Nevertheless, it is appropriate to briefly consider the role of opioid analgesics in cancer pain management. The Global Opioid Policy Initiative (GOPI), in collaboration with the Union for International Cancer Control (UICC), the Pain and Policy Studies Group (PPSG) of the University of Wisconsin in the United States, WHO, and 17 international cancer and palliative care specialty societies has evaluated the accessibility of opioids to various nations, including those of Latin America for the treatment of cancer pain. Laws enacted to help reduce the misuse or diversion of opioids may sometimes have unintended consequences by making it very difficult, inconvenient, and complex for physicians to prescribe opioids and for patients to fill prescriptions at pharmacies. Sometimes family members must return every day or every few days to pharmacies to get frequent refills, because the law may drastically restrict how many doses can be dispensed at any one time. Patients may have to register with federal authorities in order to have an opioid dispensed. Laws intended to limit overprescribing can sometimes be so strict that clinicians drastically under-prescribe opioids or refuse to prescribe opioids at all in order to avoid problems with the authorities [122].

In 2010, a research initiative by the WHO confirmed that roughly two-thirds of the global population did not consume any strong opioids for any purpose at all [123]. That means even in the most extreme situation of very severe cancer pain at end of life, the majority of people on earth have no access to adequate analgesia. In Latin America, Argentina and Brazil utilize the most opioids per capita for analgesia. Argentina, Brazil, Cuba, Mexico, and Peru make immediate-release opioids available to some cancer patients, but there is limited to no access to extended-release formulations [124]. In a meta-analysis of palliation in Latin America, morphine was the only opioid analgesic mentioned by name and often had negative cultural associations with hastening death [35]. There can also be cultural stigmatization of the use of opioids in general along with fears of addiction or tolerance.

Cancer is prevalent in Latin America with approximately 1.5 million new cases diagnosed in 2022 [125]. Demographics no doubt play a role; as the population in Latin America has doubled in the past half-century, life expectancy has increased, and cancer rates are going up. Higher rates of colorectal cancer in Latin America are attributed to lifestyle changes, greater consumption of processed foods, and an increasingly sedentary lifestyle. The aging population in the next two decades is expected to increase the Latin American cancer burden. It has been suggested that the COVID-19 pandemic has exacerbated this challenge by diverting limited healthcare resources away from cancer screenings and cancer care [125].

Cancer pain is likewise prevalent and may occur in the early as well as late stages of the disease [126]. Better pain assessment and clinician education are needed to help manage pain in this large and growing patient population.

## Conclusions

The incidence of cancer in Latin America is high and increasing, which brings with it a need for pain control and, therefore, pain assessment. Many pain assessment metrics exist and have been validated in Spanish and Portuguese, but there is no one “gold standard” instrument. Regular and accurate pain assessments are crucial to good pain care, and pain assessment need not be expensive or time-consuming. In addition to assessing pain intensity, other measurement tools are available to evaluate quality of life, types of pain, and psychosocial factors that can influence pain perception.
